# Inhibitory activity of flaxseed oil against CdCl_2_ induced liver and kidney damage: Histopathology, genotoxicity, and gene expression study

**DOI:** 10.1016/j.toxrep.2020.08.023

**Published:** 2020-08-31

**Authors:** Kawthar A. Diab, Noha E. Ibrahim, Maha A. Fahmy, Emad M. Hassan, Enayat A. Omara

**Affiliations:** a Genetics and Cytology Department, Genetic Engineering and Biotechnology Division, National Research Centre (NRC), 33 El-Bohouth Street, Dokki, Cairo, P.O. 12622, Egypt; b Microbial Biotechnology Department, Genetic Engineering and Biotechnology Division, National Research Centre (NRC), 33 El-Bohouth Street, Dokki, Cairo, P.O. 12622, Egypt; c Medicinal and Aromatic Plants Research Department, Pharmaceutical Industries Research Division, National Research Centre (NRC), 33 El-Bohouth St, Dokki, Cairo, P.O. 12622, Egypt; d Pathology Department, Medical Research Division, National Research Centre (NRC), 33 El-Bohouth Street, Dokki, Cairo, P.O. 12622, Egypt

**Keywords:** Cadmium chloride, Comet assay, Flaxseed oil, Gas-chromatograph-mass spectrometry, Gene expression of caspase-9, TNF-α, and p53, Genotoxicity, Immunohistochemistry, qRT-PCR

## Abstract

The present work evaluated the effect of flaxseed oil (FO) against toxicity induced by cadmium chloride (CdCl_2_) in the mouse liver and kidney. Male Swiss albino mice were treated with CdCl_2_ (4.5 mg/kg, intraperitoneally) with or without FO at three concentrations (4, 8, 12 mL/kg, orally) for two consecutive weeks. To analyze the effects of FO, we used the following techniques: (1) histopathological examination; (2) comet assay; (3) RT-PCR gene expression analysis of tumor necrosis factor (TNF-α) and tumor suppressor protein (p53); and (4) immunohistochemical analysis of caspase-9 protein expression. The gas chromatography-mass spectrometry results showed that FO had a high content of unsaturated fatty acids including, oleic acid, linolenic acid, and linoleic acid. Oral supplementation with FO (12 mL/kg) resulted in a normal histological appearance without alteration in the DNA integrity and gene expression of TNF-α, p53, and caspase-9 in liver and kidney tissues. As expected, CdCl_2_ remarkably induced loss of histological integrity, increased DNA comet formation, increased TNF-α and p53 mRNA expression levels and increased the immunoreactivity of caspase-9 expression. When FO was given before administration of CdCl_2_, these histopathological defects were reversed; necrosis, degeneration, inflammatory cell infiltration, hemorrhage, Kupffer cells, and pyknotic cells were all reduced. These histological improvements induced by FO were accompanied by reduced DNA breakage, downregulated mRNA expression of TNF-α and p53, and downregulated immunohistochemical expression of caspase-9 protein. In conclusion, FO and its constituents may act as signaling molecules and modify the expression of genes involved in proinflammatory cytokine production (TNF-α), cell cycle arrest (p53), and apoptosis (caspase-9), thereby improving biological activities and health.

## Highlights

• CdCl_2_ induced severe histological and genetic lesions in mouse liver and kidney.
• Flaxseed oil successfully repaired hepatic and renal histological lesions induced by CdCl_2_.
• Flaxseed oil effectively decreased comet tail formation induced by CdCl_2_ in mouse liver and kidney.
• Flaxseed oil significantly downregulated relative mRNA expression levels of TNF-α and p53 in mouse liver.
• Flaxseed oil remarkably downregulated immunohistochemical expression of caspase-9 in liver and kidney.

## Graphical abstract

**Figure:**
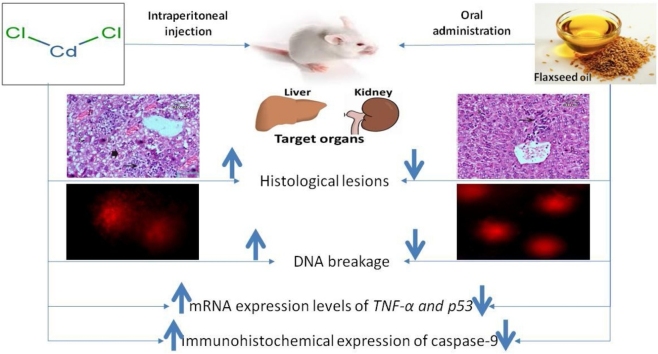

## 1 Introduction

Cadmium (Cd), an inorganic metal, exists naturally in the Earth’s crust at approximately 0.1 part per million. It frequently exists as a divalent cation and complexes with other elements (e.g., chloride, sulfide). However, manufacturing, farming, mining, and volcanic eruptions can cause an increase in the concentration of Cd in the environment [1,2]. Cd is used in various industrial applications as follows: (1) as an active electrode material in nickel-cadmium batteries; (2) as an anti-friction and anti-corrosion agent in metal coatings; (3) as a plastic stabilizer against light and heat in polyvinyl chloride; (4) as a neutron absorber in nuclear power reactors; (5) as a color pigment in paints, plastic, ceramics, and glass; and (6) as a component in various specialized alloys [3]. The most important sources of human exposure to Cd are cigarette smoking and contamination of agricultural soil with Cd-containing phosphate fertilizers and sewage sludge. Several reports have revealed a close association between Cd exposure and the incidence of severe health problems, including cancer, renal dysfunction, liver damage, osteoporosis, and cardiovascular disease [4].

Toxicity occurs when Cd accumulates inside the tissues, especially in the liver and kidney. The accumulation of Cd depends on the following factors: (1) the chemical properties of cadmium salts (chloride, sulfate, oxide, and sulfide); (2) the presence of binding sites inside the tissues; (3) ability of Cd to bind to ligands within the cells; (4) the route of administration, the dosage and the treatment period; and (5) the sensitivity of the mammalian species exposed, which is dependent upon age, sex, and race [5].

In terms of toxicokinetics, Cd is not directly metabolized in the same way as organic compounds, i.e, via oxidation, reduction or alkylation. In the liver, Cd binds to metallothionein (MT), a low-molecular scavenger protein, forming a Cd–MT complex. This complex is stored in the liver and transported around the entire body via the bloodstream. A fraction of absorbed Cd binds to thiol-containing amino acids and glutathione to maintain cellular redox balance. In the kidney, Cd–MT complexes are filtered in the renal glomeruli and reabsorbed in the renal tubules, but remain in the tubule cells. When the liver and kidney are saturated with Cd–MT, the extra Cd ions cause cellular damage in those tissues [6,7]. Unbound Cd ions enter the cells through calcium channels, interfere with mitochondrial function and stimulate the formation of reactive oxygen and nitrogen species (ROS/RNS). Consequently, Cd induces oxidative stress, lysosomal damage, lipid peroxidation, DNA breakage, DNA repair defects, abnormal gene expression, and apoptosis [2,4,8].

Recently, unsaturated fatty acids particularly polyunsaturated fatty acids (PUFAs) have been attracted much attention due to their profound biological activities. The extraction of fatty acids, either as crude extract or as pure compounds, is essential to understand their therapeutic value [9]. These fatty acids are regularly combined with glycerol-producing triglycerides. Flax *(Linum usitatissimum* L.*)*, a blue-flowering annual plant belonging to the genus *Linum* in the Linaceae family, is cultivated for its seeds and fibers. Flaxseeds, also known as linseeds, are small flat seeds varying in color from golden yellow to reddish-brown and have a brittle texture and a nutty taste. Flaxseed oil (FO) is an excellent source of PUFA that are not synthesized in the human body but are required for metabolism [10]. PUFAs are transported across the intestinal cell membrane and metabolized into longer-chain fatty acids of 20 and 22 carbon atoms. Linolenic acid, an omega-3 PUFA, is converted into a series of omega-3 PUFAs including eicosapentaenoic acid (EPA) and docosahexaenoic acid (DHA). Linoleic acid, an omega-6 PUFA, is metabolized into arachidonic acid [11].

The beneficial effects of FO and its constituents have been studied in *in vivo* and *in vitro* systems [[12], [13], [14]]. Consumption of FO for 28-days improved the lipid and glucose profile, reduced cardiac, hepatic and renal markers, reduced oxidative damage and stimulated the antioxidant defense system in patients with metabolic syndrome [15]. Recently, treatment with FO reduced cell proliferation in several human cancers and reduced tumor growth in an ectopic cervical cancer mouse model [16,17]. Although the prophylactic activity of FO has been demonstrated, its protective mechanism is complex and still incompletely understood. Thus, the present investigation was planned to explore the protective mechanism by which FO counteracts the toxic effect of CdCl_2_ in the mouse liver and kidney.

## 2 Materials and methods

### 2.1 Plant material and lipid extraction

Golden flaxseeds *Linum usitatissimum* (200 g) were purchased from a local market (Dokki, Cairo, Egypt). The seeds were deposited in the herbarium of the National Research Centre under voucher number L156. Total lipid extraction was performed to determine the fatty acid and unsaponifiable composition of the seeds. The chopped seeds were extracted with hexane (5X, 400 mL) until exhaustion at room temperature. The solvent was evaporated in a vacuum at 45 °C, and the collected oil sample (62 mL) was kept cool.

#### 2.1.1 Separation of fatty acids and unsaponifiable matter

An aliquot of the lipid matter (0.25 g) was saponified with 20 mL of methanolic potassium hydroxide (10 %) at 80 °C for three hours under reflux. The unsaponifiable matter was extracted with ether (10X, 10 mL), washed several times with distilled water, and dried over anhydrous sodium sulfate. Then, the solvent was evaporated, and the unsaponifiable matter was weighed and kept for further analysis. The lower aqueous layer (the soap solution) was acidified with 10 % HCl. The liberated fatty acids were extracted with ether (3X, 30 mL), washed several times with distilled water until acid-free and dried over anhydrous sodium sulfate. The solvent was then evaporated, and the fatty acids were weighed [18].

### 2.2 Preparation of fatty acid methyl esters

The fatty acids composition of the seeds was determined by the transesterification of the triacylglycerols in the oil to produce their respective methyl esters. Fatty acids methyl esters (FAMEs) were prepared by refluxing the liberated fatty acids (10 mg) with 2% H_2_SO_4_ (10 mL) in anhydrous methanol in a water bath for 5 h at 90 °C [19]. FAMEs were extracted with petroleum ether (10 mL each). The petroleum ether extract was treated with a dilute sodium bicarbonate solution to remove the acidity. Then, the extract was washed several times with distilled water, dried over anhydrous sodium sulfate, filtered and concentrated under reduced pressure.

### 2.3 Gas chromatography coupled with mass spectrometry

The FAME samples and unsaponifiable matter were analyzed using a gas chromatography-mass spectrometry (GC–MS) instrument with the following specifications. Instrument: a TRACE GC Ultra Gas Chromatograph (THERMO Scientific Corp., USA), coupled with a THERMO mass spectrometer detector (ISQ Single Quadrupole Mass Spectrometer). The GC–MS system was equipped with a TR-5MS column (30 m ×0.32 mm internal diameter, 0.25 μm film thickness). Analyses were carried out using helium as carrier gas at a flow rate of 0.8 mL/min at a split ratio of 1:10.

#### 2.3.1 GC–MS temperature programming

The temperature program used to determine the fatty acids profile was as follows: 80 °C for 1 min; rising at 4 °C/min to 300 °C and holding for 1 min. To determine the volatile compounds in the unsaponifiable matter, the following temperature program was used: 50 °C for 3 min; rising at 5 °C/min to 300 °C and holding for 15 min. These volatile compounds are important contributors of flavors and belong to numerous structure classes such as hydrocarbons and fatty alcohols. The injector and detector were held at 220 and 200 °C, respectively. Diluted samples (1:10 hexane, v/v) of 1 μL of the mixture was always injected. Mass spectra were obtained by electron ionization at 70 eV, using a spectral range of *m/z* 40−450. Most of the compounds were identified based on retention time and mass spectra (authentic chemicals and Wiley spectral library collection).

### 2.4 Animals

Healthy adult male Swiss albino mice (*Mus musculu*s, 3 months old, weighing 23 ± 2 g) were obtained from the animal house colony at the National Research Centre (Dokki, Cairo, Egypt). Mice were housed in polycarbonate cages containing wood chip bedding. The animals were maintained under controlled laboratory conditions (temperature of 25 ± 3 °C, 12 h light/12 h dark cycles, relative humidity of 50 % ± 15 %, standard pellet diet, and water *ad-libitum*). The study protocol was approved by the Medical Research Ethics Committee for Care and Use of Laboratory Animals of the National Research Centre as part of the project protocol under registration no. 16,210

### 2.5 Treatment schedule

Thirty mice were randomly assigned to one of six equal groups (5 mice/ group) as follows:

**Group 1:** Negative control group given distilled water

**Group 2:** Plant extract group given orally FO (12 mL/kg)

**Group 3:** Positive control group intraperitoneally injected with CdCl_2_ (4.5 mg/kg, dissolved in distilled water) for two consecutive weeks. This dose was selected according to genotoxic studies of CdCl_2_ in mice [20].

**Groups 4−6:** Three co-treated groups given FO orally (4, 8, 12 mL/kg equivalent to 0.1, 0.2 and 0.3 mL/mouse respectively) 1 h before CdCl_2_ injection (4.5 mg/kg) for two consecutive weeks. The FO dosage was selected according to previous studies [21,22].

Mice were killed by cervical dislocation 24 h after the end of the experiment. Liver and kidney tissues were collected from each group for histological and genetic analyses.

### 2.6 Histopathology

The liver and kidney tissues were excised and fixed in neutral buffered formalin (10 %). The organs were routinely processed and sectioned at 5 mm thickness using a rotary microtome. The obtained sections were placed on glass slides, deparaffinized and stained with hematoxylin and eosin stain. As described by Meyerholz and Beck [23], the severity of histological features was scored in a semi-quantitative manner as follows: none (grade 0; ≤ 5% of tissue); mild (grade 1;≤ 25 % of tissue); moderate (grade 2; ≤ 50 % of tissue); severe (grade 3; 51%–74% of tissue); and very severe (grade 4; ≥ 75 % of tissue).

### 2.7 Genotoxicity

DNA damage was evaluated by the alkaline version of single-cell electrophoresis (comet assay, pH > 13) as previously described [24]. Briefly, fresh liver and kidney tissues were washed and homogenized in phosphate buffer solution (PBS) containing 20 mM EDTA (pH 7.5). The cell suspension (10 μL, 5 × 10^6^ cells/mL) was mixed with 100 μL of low-melting agarose (0.8 %). The cell suspension was pipetted onto fully-frosted slides pre-coated with 1% normal-melting agarose to form microgels and allowed to solidify at 4 °C. The slides were dipped in chilled lysis buffer (2.5 M NaCl, 100 mM EDTA, 10 mM Tris−HCl, pH 10, containing 1% Triton X-100 and 10 % DMSO) for 2 h in the dark. Subsequently, the slides were washed with distilled water to remove the detergent from the microgels. The slides were placed in chilled alkaline buffer (300 mM NaOH and 1 mM EDTA, pH > 13.0) for 20 min, then electrophoresis was conducted at 300 mA for 30 min. The slides were neutralized in Tris-buffer (0.4 M Tris HCl, pH 7.5) twice for 5 min each time and dehydrated in absolute ethanol. The air-dried slides were stained with ethidium bromide and immediately visualized at 400× magnification using a fluorescent microscope equipped with a digital camera and green filter. One hundred nucleoids per mouse were scored using Tritek cometscore™ software (version 1.5, TriTek Corp, Sumerduck, VA 22742, USA). The percentage of DNA in the comet tail is considered to be the most reliable parameter that can be used to quantify DNA damage.

### 2.8 Gene expression analysis by real time RT-qPCR

Quantitative analysis of mRNA expression was performed by real-time RT-qPCR. Total RNA was isolated from liver tissues frozen at −80 °C using the standard TRIzol extraction method (Invitrogen, Paisley, UK) according to the manufacturer's protocol. The total RNA samples were pretreated using the DNA-free™ DNase treatment and removal reagents kit (Ambion, Austin, TX, USA) to remove any possible genomic DNA contamination. The isolated RNA was recovered in 100 μL molecular biology grade water and stored at −20 °C. The concentration and purity of the isolated RNA were determined by NanoDrop Spectrophotometer absorption (Thermo Scientific, USA) at 260 nm. First-strand cDNA synthesis performed with the total RNA sample using the SuperScript Choice System (Life Technologies, Breda, Netherlands). The qRT-PCR was carried out with 5 μL of the first-strand cDNA containing 12.5 mL 2X SYBR Green PCR Master Mix (Applied Biosystems, Foster City, CA) and 200 ng of each primer in a total volume of 25 mL. The sequences of the specific primers used are listed in Table 1. The following thermal cycling conditions were applied: 50 °C for 2 min, then 95 °C for10 min followed by 40 cycles of 95 °C for 30 s, 60 °C for 30 s, and 72 °C for 30 s. Changes in the expression of each target mRNAs were normalized relative to the mean critical threshold values of GAPDH mRNA(internal reference) using the ΔΔCt method [25].

**Table 1:** List of primers used for real-time RT-qPCR analysis.

Gene name	Gene symbol	Sequence size (bp)	Primer sequences (5′−-3)		Genebank *accession no.*
Tumor necrosis factor alpha	*TNF-α*	372 − 553	F	CAT GCG TCC AGC TGA CTA AA	AB039224.1
			R	TCC CCT TCA TCT TCC TCC TT	
Tumor suppressor protein	*p53*	882 − 1113	F	AGA GAC CGC CGT ACA GAA GA	M13874.1
			R	CTG TAG CAT GGG CAT CCT TT	
Glyceraldehyde 3-phosphate dehydrogenase	*GAPDH*	1725 − 1913	F	CGG CTA CTA GCG GTT TTA CG	AY340484.1
			R	AAG AAG ATG CGG CTG ACT GT	

### 2.9 Immunohistochemical expression of caspase-9

Liver and kidney sections were deparaffinized and pretreated with 10 mM citrate buffer. The slides were washed with PBS and incubated overnight at 4 °C in a humidified chamber with mouse monoclonal antibody to caspase-9 (1:100 dilution, Acris Antibodies, Hiddenhausen, Germany). Then, the sections were rinsed again with PBS and incubated with a biotinylated goat anti-rabbit immunoglobulin G secondary antibody for 10 min at room temperature. Finally, the sections were incubated with streptavidin peroxidase. To visualize the immunoreactivity reaction product, the slides were incubated with 3, 3′-diaminobenzidine tetrahydrochloride (DAB, Sigma-Aldrich, USA) for 10 min. The slides were counterstained with hematoxylin, dehydrated, and mounted. Primary antibodies were omitted and replaced with PBS for negative controls.

### 2.10 Statistical analysis

Data were analyzed using the computerized software SPSS (Statistical Package of Social Science, version 20, Armonk, New York: IBM Corp). One-way analysis of variance (ANOVA) followed by Tukey's multiple comparisons test was used to analyze the differences among the experimental groups. The level of statistical significance was set at P <0.05.

## 3 Results

### 3.1 Fatty acid profile of FO

The fatty acid profile of FO was analyzed as methyl ester using gas-liquid chromatography. Fatty acids consist of a long-chain hydrocarbon with a carboxylic acid (R−COOH) and a methyl group (CH3) at the start and the end of the chain, respectively. Fatty acids are classified as according to the number of double bonds in the chain: (1) monounsaturated fatty acids (MUFAs, single double bond), (2) polyunsaturated fatty acids (PUFAs, ≥ 2 double bonds) and (3) saturated fatty acids (SFAs, no double bonds). As shown in Table 2, the lipid fraction of FO had an ideal balance of MUFAs to PUFAs and SFAs. The concentration of oleic acid (MUFA omega-9) was highest, followed by linolenic acid (PUFA omega-3), then linoleic acid (PUMF omega-6) and finally SFAs (palmitic acid and stearic acid).

**Table 2:** GC–MS analysis of flaxseed fatty acid methyl esters.

Fatty acids	Retention time (min)	Concentration (%)
**Mnounsaturated fatty acids (MUFA)**		
Oleic acid, methyl ester	32.34	50.03
**Polyunsaturated fatty acids (PUFAs)**		
Linoleic acid, methyl ester (omega-6)	32.11	8.96
Linolenic acid, methyl ester (omega-3)	32.32	35.14
**Saturated fatty acid (SFAs)**		
Palmitic acid, methyl ester	28.1	4.52
Stearic acid, methyl ester	32.86	1.20

### 3.2 Chemical composition of unsaponifiable matter

As shown in Table 3, the unsaponifiable matter in FO is composed of eighteen saturated hydrocarbons, one unsaturated hydrocarbon, and four fatty alcohols. These compounds resulted from the decarboxylation and bioremediation of fatty acids.

**Table 3:** GC–MS analysis of unsaponifiable matter in FO.

Compounds	Retention time (min)	Concentration (%)
**Unsaturated hydrocarbon**		
1-Octadecyne	10.31	0.97
**Saturated hydrocarbons**		
2,3-Dimethyldecane	10.49	1.07
Dodecane	11.67	13.17
Undecane, 2,6-dimethyl	12.02	5.41
Cyclohexane, 2-butyl-1,1,3-trimethyl-	12.32	1.61
18-Nonadecen-1-ol	13.02	1.47
2,3-Dimethylundecane	13.55	2.73
Nonadecane	13.74	3.72
Farnesane	13.93	9.64
5-Tetradecene	14.06	2.12
Tridecane	14.95	30.12
Nonadecane	15.42	1.72
Octadecane, 1-chloro	16.51	2.68
Tetradecane, 2,610-trimethyl-	16.61	1.14
Tetradecane, 6,9-dimethyl	16.78	1.16
10-Methylnonadecane	16.98	2.09
Tridecane, 3-methyl-	17.19	1.11
Phytane	17.28	2.75
Tetradecanet	18.15	8.46
**Fatty alcohols**		
2-Butyl-1-octanol	10.69	1.66
1-Decanol, 2-hexyl-	13.31	1.57
2-Hexyl-1-octanol	13.37	2.21
1-Eicosanol	14.58	1.42

### 3.3 Histopathological effect of CdCl_2_ and FO

**Fig. 1:**
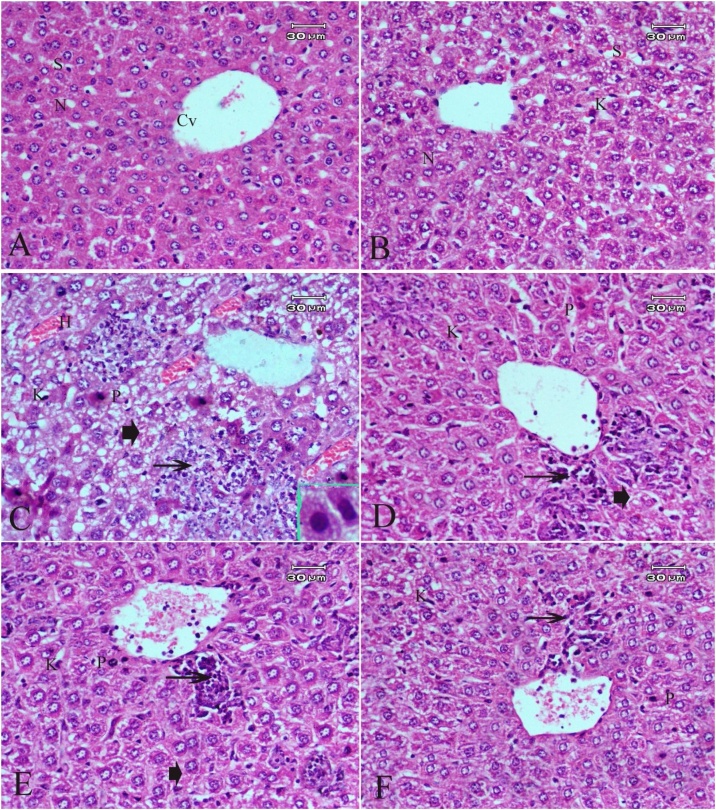
Photomicrographs of liver sections stained with hematoxylin and eosin stain (original magnification 400×) showing: **(A)** Control group ; **(B)** FO-treated group;**(C)**CdCl_2_-treated group; **(D)** Group treated with CdCl_2_ and FO (4 mL /kg); **(E)** Group treated with CdCl_2_ and FO (8 mL /kg);**(F)** Group treated with CdCl_2_ and FO (12 mL /kg); Central vein (CV); (S) Sinusoids; Nucleus (N); Pyknotic nuclei (P); Kupffer cells (K); Degeneration or necrosis of hepatocytes (arrowhead), Inflammatory cell infiltration (arrow).

**Fig. 2:**
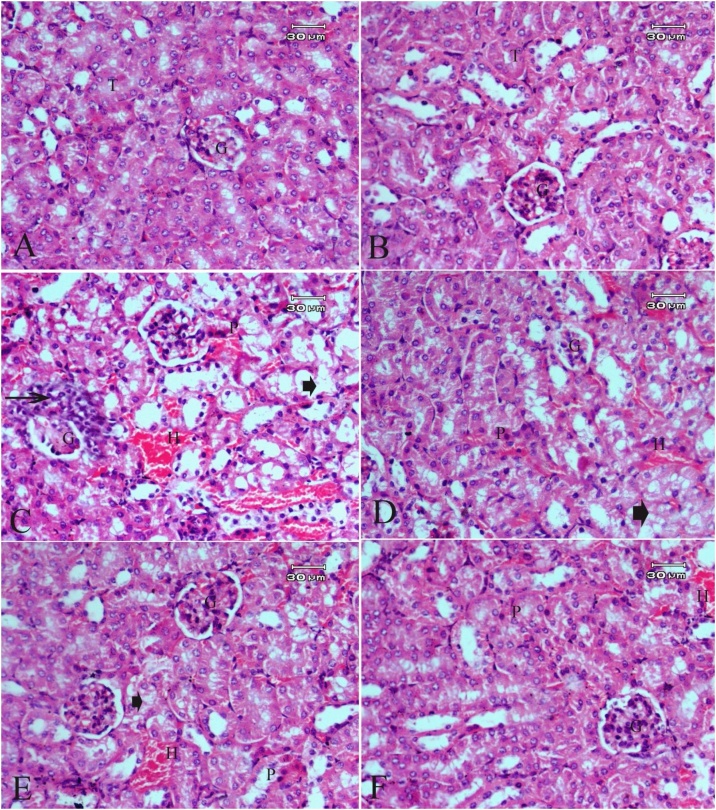
Photomicrographs of kidney sections stained with hematoxylin and eosin stain (original magnification 400×) showing **(A)** Control group ; **(B)** FO-treated group;**(C)**CdCl_2_-treated group**; (D)** Group treated with CdCl_2_ and FO (4 mL/kg); **(E)** Group treated with CdCl_2_ and FO (8 mL /kg); **(F)**Group treated with CdCl_2_ and FO (12 mL/kg); Glomerulus (G), Tubules (T); Pyknotic nuclei (P); Intracellular hemorrhage (H); Periglomerular inflammatory cells (arrow),Cytoplasmic degeneration of cells of the renal tubules (arrowhead).

Table 4, Table 5 and Fig. 1, Fig. 2 describe the histopathology findings regarding CdCl_2_ and FO. As expected, CdCl_2_ caused severe histological abnormalities in the liver and kidney sections compared to the normal histological architectures of the tissues in the control group and FO-treated group. These histological deformities were gradually recovered in a concentration-dependent manner when three concentrations of FO were simultaneously administrated alongside CdCl_2_.

**Table 4:** Histopathological observation of liver sections after treatment with CdCl_2_ and FO.

Treatment	Severity of abnormalities	Histological features
Control	Grade (0)	▪ Normal structure with radial arrangement of hepatocytes around the central vein and prominent nucleus (Fig. 1A)
FO only	Grade (0)	▪ Normal histological appearance with few dilated blood sinusoids and Kupffer cells (Fig. 1B)
CdCl_2_ only	Grade (4)	▪ Disarrangement of hepatic strands ▪ Focal area of hepatocellular necrosis ▪ Dilation and congestion of blood vessels ▪ Degeneration of hepatocytes ▪ Large hemorrhagic areas ▪ Dense inflammatory cell infiltration around the central vein ▪ Activated Kupffer cells ▪ Pyknotic nuclei or dark stained condensed nuclei (Fig. 1C).
CdCl_2_ + FO (4 mL/kg)	Grade (3)	▪ Mild disarrangement of hepatic strands ▪ Less inflammatory cell infiltration ▪ Kupffer cells ▪ Degeneration of hepatocytes (Fig. 1D)
CdCl_2_ + FO (8 mL/kg)	Grade (2)	▪ Absence of hemorrhage ▪ Mild lymphocytic infiltration around the central vein ▪ Inflammatory cell infiltration ▪ Mild necrotic areas ▪ Kupffer cells ▪ Pyknotic nuclei (Fig. 1E)
CdCl_2_ + FO (12 mL/kg)	Grade (1)	▪ Necrosis rare ▪ Little inflammatory cell infiltration ▪ Kupffer cells ▪ Pyknotic nuclei (Fig. 1F)

**Table 5:** Histopathological observation of kidney sections after treatment with CdCl_2_ and FO.

Treatment	Severity of abnormalities	Histological features
Control	Grade (0)	▪ Normal histological structure of the glomerulus and tubules (Fig. 2A).
FO only	Grade (0)	▪ Normal architecture of kidney (Fig. 2B).
CdCl_2_ only	Grade (3)	▪ Shrinking or degeneration of the glomerular tuft ▪ Periglomerular inflammatory cells ▪ Widening of the urinary space ▪ Renal cell cytoplasmic degeneration ▪ Pyknotic nuclei ▪ Intracellular hemorrhage ▪ Necrosis in some tubules (Fig. 2C).
CdCl_2_ + FO (4 mL/kg)	Grade (2)	▪ Partial improvement in glomeruli and the renal tubules ▪ Foci of intracellular hemorrhage in the cells of renal tubules ▪ Some pyknotic nuclei ▪ Degeneration of tubules (Fig. 2D, E).
CdCl_2_ + FO (8 mL/kg)		
CdCl_2_ + FO (12 mL/kg)	Grade (1)	▪ No signs of inflammation ▪ Intracellular hemorrhage ▪ Few pyknotic nuclei (Fig. 2F).

### 3.4 Effect of CdCl_2_ and FO on DNA breakage

**Fig. 3:**
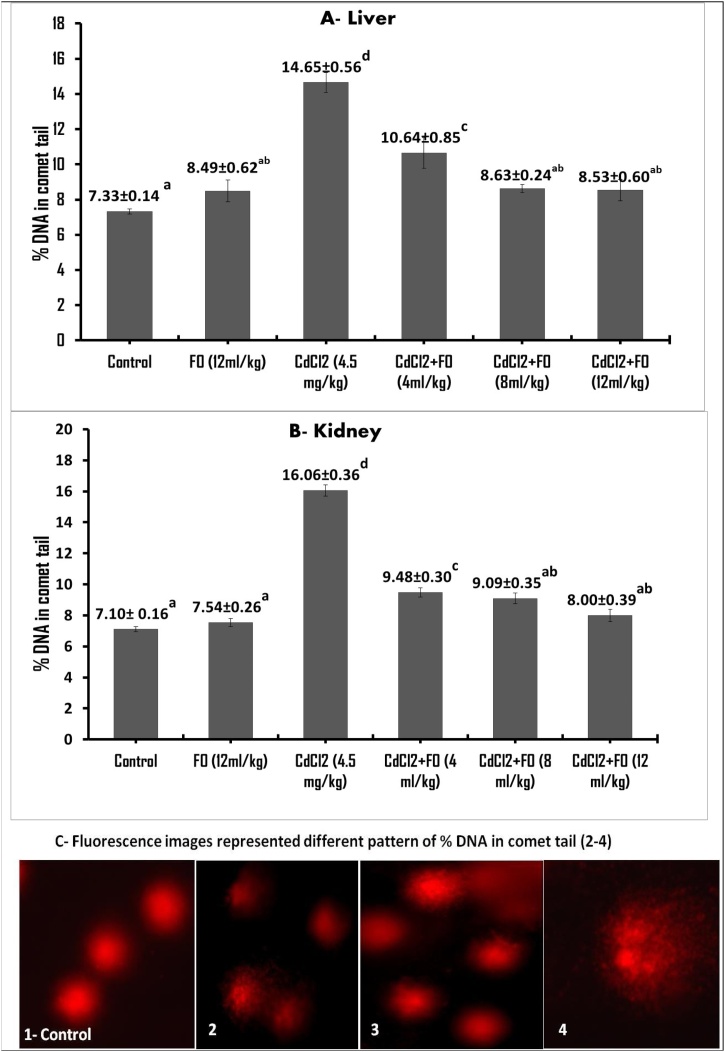
DNA-strand breakage as quantified by alkaline comet assay in the mouse liver and kidney after treatment with CdCl_2_ and FO. Data presented as mean% ± S.E (n=5). Total 500 cells per each group were analyzed using Tritek cometscore™ software. The means that carry dissimilar superscript letters are statistically significant different (P<0.05). The values that carry the similar superscript letters are not statistically significant different (P> 0.05).

To explore the inhibitory activity of FO on CdCl_2_-induced genotoxicity, DNA-strand breakages in mouse livers and kidneys were examined using a comet assay. As shown in Fig. 3, the DNA-strand breaks in the damaged cells migrated toward the anode, forming a comet tail during electrophoresis. As shown in Fig. 3, FO did not induce a statistical increase in the percentage of DNA in the comet tail in the liver (8.49 % versus 7.33 % in the control) or kidney (7.54 % versus 7.10 % in the control). In contrast, CdCl_2_ remarkably increased the percentage of tail DNA in the liver (14.65 %, i.e., a 2.0-fold increase) and kidney (16.06 %, i.e., a 2.2-fold increase) compared to their control values. Co-supplementation with three concentrations of FO and CdCl_2_ significantly decreased the percentage of DNA in the comet tail in the liver (10.64 %, 8.63 %, and 8.53 %, respectively) and kidney (9.48 %, 9.09 %, and 8.00 %, respectively) compared to the CdCl_2_ only treatment group.

### 3.5 Effect of CdCl_2_ and FO on TNF-α and p53 mRNA

**Fig. 4:**
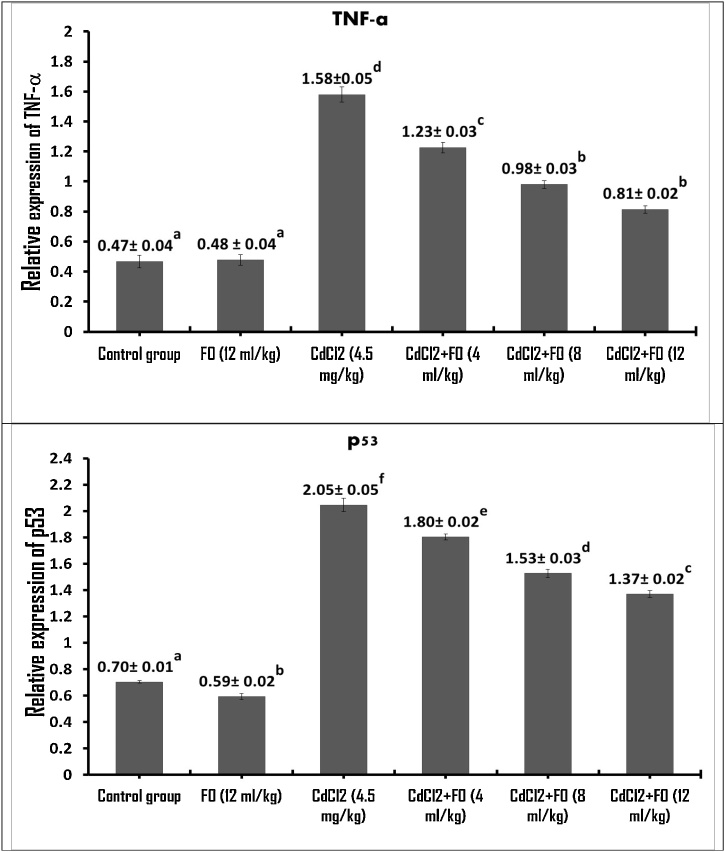
Change in gene expression of TNF-α and p53 in mouse liver as quantified by RT-qPCR after treatment with CdCl_2_ and FO. Data presented as mean% ± S.E (n=3). The means that carry dissimilar superscript letters are statistically significant different (P<0.05). The values that carry the similar superscript letters are not statistically significant different (P> 0.05).

To understand the preventive effect of FO against hepatic inflammation and apoptosis induced by CdCl_2_, TNF-α (encoding proinflammatory cytokines) and p53 (encoding pro-apoptotic protein) were examined using RT-qPCR. As shown in Fig. 4, treatment with FO alone (12 mL/kg) had no significant effect on the level of TNF-α and p53 mRNA expression in the mouse liver compared to the control group. In contrast, CdCl_2_ dramatically increased the level of TNF-α (1.58, i.e., a 3.36-fold increase) and p53 (2.81-fold increase) mRNA expression compared to control values. Treatment with both CdCl_2_ and three concentrations of FO significantly decreased the elevated relative mRNA expressions of TNF-α (1.2-, 1.6-, and 1.9-fold decrease, respectively) and p53 (1.13-, 1.33 and 1.49-fold decrease, respectively) induced by CdCl_2_ in a concentration-dependent manner.

### 3.6 Effect of CdCl_2_and FO on immunohistochemical expression

**Fig. 5:**
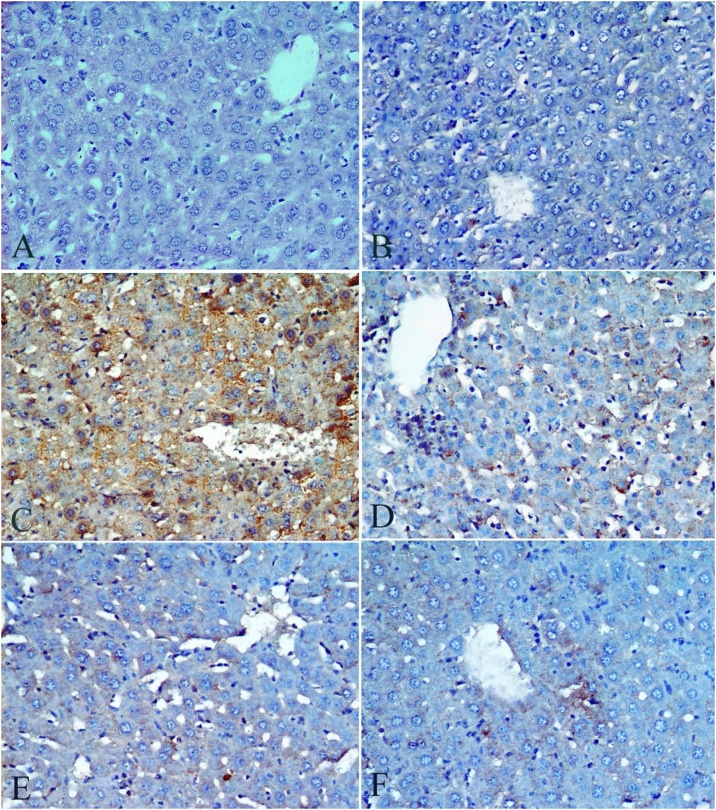
Photomicrographs of liver sections stained with caspase-9 immunohistochemical stain (original magnification 400×) showing **(A)** Control group ; **(B)** FO-treated group;**(C)** CdCl_2_-treated group**; (D)** Group treated with CdCl_2_ and FO (4 mL /kg); **(E)** Group treated with CdCl_2_ and FO (8 mL /kg);**(F)** Group treated with CdCl_2_ and FO (12 mL /kg).

**Fig. 6:**
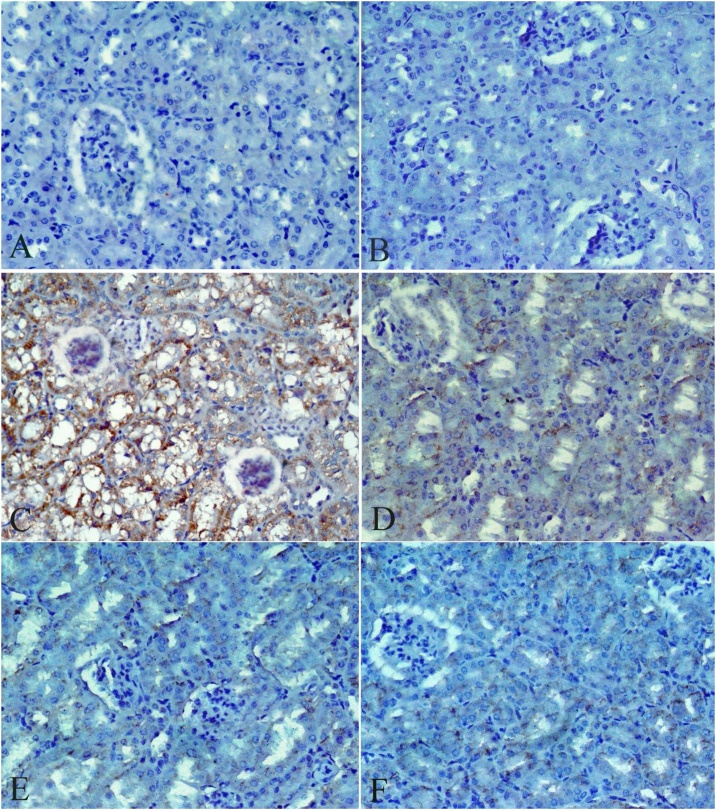
Photomicrographs of kidney sections stained with caspase-9 immunohistochemical stain (original magnification 400×) showing **(A)** Control group ;**(B)** FO-treated group;**(C)** CdCl_2_-treated group**; (D)** Group treated with CdCl_2_ and FO (4 mL/kg); **(E)** Group treated with CdCl_2_ and FO (8 mL/kg);**(F)** Group treated with CdCl_2_ and FO (12 mL/kg).

The immunoreactivity of caspase-9 (encoding apoptotic initiator caspases) was examined to clarify the probable apoptotic and anti-apoptotic pathways of CdCl_2_ and FO, respectively. Negative immunoreaction of caspase-9 expression was observed in the liver (Fig. 5A, B) and kidney (Fig. 6A, B) sections from control and FO-treated mice. In contrast, dense positive immunoreactivity of caspase-9 expression was observed in the CdCl_2_-treated group around the hepatic central vein (Fig.5 C) and proximal tubular epithelial cells and glomerulus (Fig. 6C). Interestingly, a gradual reduction in the immunoreaction of caspase-9 expression was observed in the liver and kidney section of the three co-treatment groups (FO + CdCl_2_). Three concentrations of FO (4, 8, and 12 mL/kg) produced moderate staining (Figs. 5, 6D), mild staining (Figs. 5, 6E), and a small number of positively stained cells (Figs, 5, 6 F), respectively, in both liver and kidney sections.

## 4 Discussion

In our study, FO had a high omega-3 to omega-6 ratio (4:1), suggesting that it is highly effective in reducing the risk of chronic diseases [12]. Surprisingly, FO had a lower linolenic acid content (35.14 %) and a higher oleic acid content (50.03 %) than reported by several authors. For example, Choo et al. [26], found that seven cold-pressed FOs had high linolenic acid content (58.87%–60.42%), and low oleic acid content (13.44 %–14.64 %). This variation in the FO fatty acid composition is mainly due to the extraction process and the variety of flaxseeds analyzed. Generally, golden FO has a lower linolenic acid content than brown FO [27].

The histopathological lesions in the CdCl_2_-treated group were activated Kupffer cells, inflammation, hemorrhages, pyknotic nuclei, and areas of degeneration and necrosis. These observations support the upregulation of the hepatic TNF-α mRNA expression level in the current report. The activation of Kupffer cells and resident liver macrophages leads to the release of inflammatory cytokines and ROS, which are responsible for CdCl_2_-induced histopathological injuries [28]. Similar hepatic and renal histopathological damage has been reported in previous studies [8,29,30].

Two-weeks of repeated oral treatment with FO successfully prevented hepatic and renal histological deformities induced by CdCl_2_ in a concentration-dependent manner. These findings imply that FO can block the inflammatory process and oxidative stress, which are responsible for CdCl_2_-induced toxicity. These observations support the down-regulation of TNF-α mRNA expression observed in the present work and are also in agreement with previous studies [21,22,[31], [32], [33]]. For example, Hendawi et al. [34], found that oral administration of FO (0.5 g/kg) inhibited thiacloprid-induced liver histopathological damage in rats by promoting antioxidant defense system, decreasing lipid peroxidation, reducing proinflammatorycytokines, and stabilizing the DNA during cell division.

In this study, two weeks of repeated intraperitoneal injection of CdCl_2_ caused DNA strand breakage in the mouse liver and kidney. These results reflect the fact that CdCl_2_ interacts indirectly with the DNA molecule in three possible ways: (1) interaction of CdCl_2_ with DNA–protein crosslinks; (2) inhibition of the DNA repair mechanism; and (3) induction of cellular immunity and oxidative stress [35]. These data support the upregulating of p53 mRNA expression in the CdCl_2_-treated group in the present study. Notably, upregulation of p53 is associated with cell cycle arrest in G1 phase due to the presence of unrepaired single-strand DNA breaks [36,37]. Previous studies have reported, based on comet assays, that cadmium induces genotoxicity in the stomach, liver, kidney, lung, blood, bone marrow, brain and testicle [8,[38], [39], [40]].

One of the most prominent findings of the present study was the pronounced reduction in the percentage of DNA in the comet tail induced by CdCl_2_ in the mouse liver tissues upon FO administration. These data indicate that FO can interfere with CdCl_2_ before it induces DNA strand breaks. This may occur through several mechanisms: (1) FO scavenges free radicals to terminate the formation of hydroperoxides, (2) FO chelates transition metals to suppress the generation of radical formation or decompose lipid peroxides; or (3) FO reduces the interaction of CdCl_2_ with lipid bilayers, thus preserving the integrity of the cell membrane [41]. This is in agreement with the findings of Abdel Moneim et al. [22], who found that oral administration of FO (1000 mg/kg) prevented lead acetate–induced DNA fragmentation (agarose ladder) in rat brain. Furthermore, *in vivo* and *in vitro* studies have demonstrated the antigenotoxicity of PUFAs against cyclophosphamide [42], imazalil fungicide, [14], thiacloprid insecticide [34] and heavy metals [13,43] using analysis of chromosomal aberrations and micronucleus assays.

To further understand the molecular pathway of the inhibitory activity of FO on CdCl_2_-induced toxicity, we explored the gene expression profile of TNF-α, p53, and caspase-9. Our experiments showed that CdCl_2_ increased the relative mRNA expression of TNF-α and p53 and increased the immunoreactivity of caspase-9 expression. These observations suggest that CdCl_2_ activated p53, causing the release of cytochrome c from the mitochondria into the cytosol. The binding of cytochrome c with caspase-9 resulted in the formation of apoptosomes and the activation of caspase-3, which in turn triggered apoptosis [44]. This is agreement with the findings of previous studies. For example, Miltonprabu et al. [8] reported that CdCl_2_ increased comet tail formation and increased the expression of proinflammatory cytokines (TNF-α) and apoptotic signaling protein (caspase 3, caspase 9, and cytochrome c) in rat liver. Further, Mohamed [45] found that CdCl_2_ induced mutation in exon 7 of p53, leading to upregulating of p53 immunohistochemical expression and increasing apoptotic DNA damage and degeneration process in the livers, kidneys, and brains of male rats.

Importantly, co-supplementation with FO in the present study markedly downregulated the expression of TNF-α, p53, and caspase-9 in the three co-treated groups compared to the CdCl_2_ only group. These results imply that unsaturated fatty acids can alter the composition of the phospholipids of the cell membrane and can act as precursors of signaling molecules and ligands of nuclear receptors. They can stabilize the mitochondrial membrane, preventing cytochrome c release, caspase activation, and ROS formation [31,46]. Further, linolenic acid and its derivatives (EPA & DHA) are metabolized into eicosanoids, the prostaglandin E_2_ series, and leukotriene B_4_, which are responsible for downregulation of inflammatory responses [47]. In this way, administration of FO dramatically reversed the elevated level of TNF-α- induced by alcohol in the mouse liver [32], and the pesticide thiacloprid in the rat liver [34]. Treatment with EPA caused downregulation of p53 immunohistochemical expression in UAV-irradiated human skin [48]. Supplementation with EPA effectively reduced caspase-mitochondrial apoptosis (regulated by cleaved caspase-3, caspase-9, and cytochrome c) in the EPA-treated diabetic mouse kidney [46].

It is plausible that limitations may have influenced the findings obtained in the present study. Due to the financial limitation of in-house research projects in developing countries, we did not evaluate the concentration of Cd inside the tissues. Additionally, we were not able to assess biochemical functions and responses in the blood and tissues such as liver and kidney function; and oxidative stress. Despite these limitations, our findings suggest that FO may alleviate CdCl_2_-induced histopathological deformities in the liver and kidney through the following actions: (1) reduction of the percentage DNA breaks in liver and kidney tissues; (2) downregulation of hepatic mRNA expression of TNF-α, and p53; and (3) downregulation of caspase-9 expression using immunohistochemical staining. However, further studies using more precise biomarkers are needed to better understand the molecular protective mechanism of FO and its constituents.

## CRediT authorship contribution statement

**Kawthar A. Diab:** Investigation, Visualization, Writing - review & editing. **Noha E. Ibrahim:** Investigation, Visualization. **Maha A. Fahmy:** Project administration, Conceptualization, Writing - review & editing. **Emad M. Hassan:** Investigation, Visualization. **Enayat A. Omara:** Investigation, Visualization.

## Declaration of Competing Interest

The authors declare that they have no known competing financial interests or personal relationships that could have appeared to influence the work reported in this paper.
